# Characterising the Hospital Microbiota: A Descriptive Study of Environmental Monitoring

**DOI:** 10.3390/pathogens15090904

**Published:** 2026-08-27

**Authors:** Antonio Laganà, Giuseppe La Spada, Gaetano Massara, Giuseppa Visalli, Adal Waqas Ali, Massimo Sartelli, Alessio Facciolà

**Affiliations:** 1 Department of Biomedical Sciences, Dental Sciences and Morpho-Functional Imaging, University of Messina, Via C. Valeria, 98125 Messina, Italy; antonio.lagana@unime.it (A.L.); giuseppe.laspada@studenti.unime.it (G.L.S.); gaetano.massara@studenti.unime.it (G.M.); giuseppa.visalli@unime.it (G.V.); 2 Department of Internal Medicine, University Hospital “Maggiore della Carità”, Corso Giuseppe Mazzini 18, 28100 Novara, Italy; awadil4991@gmail.com; 3 Department of General Surgery, Macerata Hospital, 62100 Macerata, Italy; massimosartelli@gmail.com

**Keywords:** hospital microbiota, microbiological monitoring, hospital environment contamination, ESKAPEE pathogens

## Abstract

The hospital environment is widely recognised as a source of microorganisms that collectively constitute the so-called “hospital microbiota”. The aim of this study was to investigate this microbiota, providing as accurate a picture as possible of the actual level of environmental contamination within the hospital. We conducted a 2-year surveillance study in a large acute-care hospital to map the microbiological profiles of the various wards and identify areas requiring improvement. The analysis revealed substantial contamination of surfaces by ESKAPEE microorganisms, well-known causes of healthcare-associated infections. Near-patient surfaces and some critical items such as IV poles, medication carts, and infusion pumps, were found to be contaminated. Among wards, the highest level of contamination was found in the medical area, followed by emergency and surgical wards. A substantial amount of antibiotic-resistant microorganisms was detected, including methicillin-resistant *Staphylococcus aureus*, vancomycin-resistant enterococci, multidrug-resistant *Acinetobacter baumannii* and carbapenem-resistant *Enterobacteriaceae*. The presence of these microorganisms on particularly critical surfaces and in critical wards poses a serious risk. Monitoring environmental contamination could help identify critical issues and assess the necessity of improving environmental disinfection protocols in addition to the well-known procedures for preventing healthcare-associated infections.

## 1. Introduction

Healthcare settings such as hospitals and long-term care facilities provide ideal ecological niches for resistant organisms because of high antimicrobial drug consumption, frequent invasive procedures, and overcrowding [1]. Recently, the concept of “hospital microbiota”, defined as the community of microorganisms present in the hospital environment, and their relationships with healthcare-associated infections (HAIs), has been studied in the international literature [2]. In these settings, the interaction among patient risk factors, environmental reservoirs, and healthcare practices sustains transmission chains that are challenging to interrupt once established [3].

A small but critical group of bacterial pathogens, designed in 2017 by the World Health Organisation (WHO) with the acronym ESKAPE (*Enterococcus faecium*, *Staphylococcus aureus*, *Klebsiella pneumoniae*, *Acinetobacter baumannii*, *Pseudomonas aeruginosa*, and *Enterobacter* spp.), disproportionately drives the burden of HAIs and antimicrobial resistance (AMR) in hospitals. This acronym was subsequently updated to ESKAPEE (*Enterococcus faecium*, *Staphylococcus aureus*, *Klebsiella pneumoniae*, *Acinetobacter baumannii*, *Pseudomonas aeruginosa, Enterobacter* spp. and *Escherichia coli*) [4]. These species combine multiple resistance mechanisms with traits that favour their persistence and spread in the hospital environment, including biofilm formation and prolonged survival on inanimate surfaces [5,6,7,8]. For these reasons, they are frequent causative agents of the main device- and procedure-related HAIs, such as central-line-associated bloodstream infections (CLABSIs), ventilator-associated pneumonias (VAPs), catheter-associated urinary tract infections (CAUTIs), and surgical site infections (SSIs) [9].

Many conventional and unconventional hospital surfaces can harbour ESKAPEE pathogens and could act as potential sources of HAIs. In this context, so-called high-touch surfaces play a particularly crucial role [10,11,12,13,14]. The proven survival of microbes on surfaces provides the basis for their cross-transmission between patients, healthcare workers (HCWs) and the environment, with HCWs’ and patients’ hands playing a primary role [15].

In view of the above, environmental sanitation plays a crucial role in preventing and containing HAIs [16,17]. For this reason, this procedure was categorised as one of the eight Standard Precautions for Infection Prevention and Control (IPC), along with other cornerstones of infection prevention, including hand hygiene and the use of personal protective equipment (PPE) [18]. However, despite being a crucial component of IPC, environmental hygiene can be greatly hindered by many factors, such as variability in disinfectant purchasing, the composition of surface materials and their compatibility with the chemical products used, the concentration and contact time of disinfectants, and the training/turnover/shortage of cleaning personnel [19,20,21]. Therefore, adherence to sanitation practices can vary considerably across facilities and over time, often deteriorating, especially when monitoring is poor or staffing is constrained. Consequently, monitoring adherence to cleaning protocols and assessing the efficacy of hospital sanitation can contribute greatly to managing HAIs, as highlighted by recent scientific evidence [22,23,24].

International recommendations for hospital sanitation monitoring involve different approaches, such as visual checks, adenosine triphosphate (ATP) bioluminescence, the use of fluorescent markers and microbial sampling [23,25]. Some key practices include the proper disposal of hazardous medical waste posing an infection risk (needles, sharps, and contaminated items in general), defined cleaning protocols (frequency, products, high-to-low surfaces, and specific equipment), staff training, and the establishment of national standards/accountability for consistent, high-quality environmental hygiene to prevent HAIs.

Despite all this evidence, methods for verifying environmental sanitation in healthcare facilities are not routinely integrated into performance dashboards because of challenges related to various methods that can potentially be used, a lack of human and economic resources, a lack of standardised metrics, and the complexity of linking cleaning to infection outcomes.

In the light of the above, this study was structured as a descriptive analysis characterising the hospital microbiota, with an evaluation of strains potentially able to cause HAIs on various hospital surfaces, which could represent a potential risk if the well-established good practices and rules are not followed. Accordingly, the study aims to demonstrate that microbiological monitoring of the hospital environment could represent a tool to map the environmental microbiological profile of various wards and assess infection risk by identifying areas requiring improvement, thereby complementing well-established ICP programmes.

## 2. Materials and Methods

### 2.1. Setting

We conducted a retrospective laboratory-based observational study at the University Hospital “G. Martino” in Messina, Italy, covering the 2-year period from 1 January 2024 to 31 December 2025. The research was based on the collection and analysis of environmental microbiological monitoring data from hospital surfaces. The Messina University Hospital is a large acute-care healthcare facility with approximately 600 beds for ordinary and day hospital/surgery admissions, and 24 operating rooms, encompassing all the major medical and surgical disciplines. This healthcare facility records an average of more than 30,000 hospital admissions per year. This number includes both patients admitted for ordinary admissions and those admitted to the Day Hospital and Day Surgery units across the hospital’s various departments.

### 2.2. Sampling Strategy and Site Selection

In accordance with our hospital procedures, environmental samplings are carried out by our Hospital Hygiene Operative Unit in two different ways: routinely, at regular intervals (specifically, every 2 months, alternating between operating rooms and patient rooms in the various wards) and upon specific request following the detection of an “alert” microorganism by the Clinical Microbiology Unit. These latter controls are particularly important because they are closely linked to the detection of an infection caused by pathogens internationally recognised as posing a particular risk to patients, HCWs, or the community because of their high virulence, contagiousness, and/or AMR [26]. Specifically, in operating rooms, both air and surfaces are sampled, while in wards, surfaces and HCWs’ hands are checked. The three primary outcomes are to quantify surface contamination, expressed as colony-forming units (CFUs) per 100 cm^2^ (CFU/100 cm^2^) for surfaces and CFU per 1000 L (CFU/m^3^) for air; to assess the frequency of ESKAPEE detection among isolates; and to evaluate critical issues requiring improvement, such as contamination of “critical” surfaces in “critical” settings.

We normally perform these controls to evaluate the need to implement sanitation procedures in hospital environments where necessary, and/or to trace possible chains of transmission in the event of an outbreak.

In operating rooms, surfaces are sampled according to the Italian national guideline on “Safety and Hygiene Standards in Operating Room” [27]: the surgical bed, surgical instrument table, and surgical light are routinely sampled, with additional surfaces varying according to the operating room sampled (e.g., monitors, desktops, and medication carts). Air samples are actively collected before (“at rest”) and during (“operational”) surgical activity using a sampler (SAS, Surface Air System), that actively draws in a known volume of air (500 mL/5 min). The collected air is directed onto a Plate Count Agar (PCA) plate, because the analysis is purely quantitative.

In wards, particularly high-touch and near-patient surfaces inside patient rooms are sampled (e.g., bed rails, over-bed tables, infusion pumps, door handles, call buttons, and taps) soon after sanitation procedures. In addition, surfaces in medication rooms (e.g., medication carts, taps, medicine cabinet handles) and HCWs’ hands, with or without gloves, are checked during daily caregiving activities.

### 2.3. Environmental Surface Sampling

Surface sampling is performed using contact slides and/or sterile swabs. Both materials are purchased by Liofilchem S.r.l. (Roseto degli Abruzzi, TE, Italy). Contact slides are ready-to-use double-sided devices with two different media coated onto a plastic support. Specifically, three different types of contact slides are used:

▪ Contact slide with PCA + 2,3,5-Triphenyltetrazolium Chloride (TTC) + Neutralising for total bacterial count, and Vogel–Johnson Agar for the detection of *Staphylococcus* spp.;
▪ Contact slide with Cetrimide Agar + Neutralising for the detection of *Pseudomonas* spp., and Rose Bengal Caf Agar + Neutralising for yeasts/moulds;
▪ Contact slide with Violet Red Bile Glucose Agar (VRBG agar) + Neutralising for the detection of Gram-negative bacteria, and Bile Esculin Agar (BEA) + Neutralising for *Enterococcus* spp.

For the collection technique, both sides of the slide are pressed lightly onto the surface to be examined for 10 s. The slides are then incubated at 37 °C for 48–72 h. Each side of the slide covers a 10 cm^2^ surface; therefore, the result, expressed as CFU/100 cm^2^, is multiplied by 10. Slides are particularly suitable for flat surfaces.

For the swab technique, a sterile swab pre-moistened with neutralising buffer (e.g., lecithin, polysorbate, and sodium thiosulfate) is rubbed over the surface using vertical, horizontal, and diagonal strokes with uniform pressure for approximately 10–15 s. Specifically, a 10 × 10 cm area on each surface is delimited using a sterile template. The swabs are then transported at 2–8° C to the laboratory within 2 h. After vortexing, aliquots of the eluates are plated onto the culture media detailed below. Swabs are used particularly for irregular surfaces to which contact slides could not be applied.

### 2.4. Culture Media, Incubation Conditions and Interpretation of the Results

After sampling, swabs are plated on the following media: PCA for total aerobic bacterial count, Mannitol Salt Agar (MSA) for staphylococci, MacConkey Agar (MCA) for *Enterobacterales* and other Gram-negative bacteria, Bile Esculin Azide Agar (BEAA) for enterococci, and Sabouraud Dextrose Agar (SDA) for yeasts/moulds. Each plate type is incubated under standard aerobic conditions at the appropriate temperature and duration according to the medium class; plates are read at 24 h and re-examined up to 48–72 h when indicated. For quantitative results, CFU were enumerated and normalised to CFU/100 cm^2^. Specifically, for operating rooms, positivity is established according to the guidance provided by the already cited national guidelines [27] (≥16 CFU/100 cm^2^ for total bacterial count, whereas even a single colony of an ESKAPEE microorganism is to be considered a positive result). The same guideline establishes for air samples a non-conforming result following to the detection of ≤35 CFU/m^3^ and ≤180 CFU/m^3^ for “at rest” and “operational” samplings, respectively. As regards wards, there is not a specific guideline and, as this is a qualitative descriptive study whose main aim is to reconstruct the hospital microbiota, even a single colony of a microbial strain was taken into account as an indicator of the circulation of that strain within the hospital.

### 2.5. Isolate Identification and Antimicrobial Susceptibility Testing (AST)

Representative colonies from distinct morphotypes and all presumptive ESKAPEE organisms were subcultured for identification and AST, which were performed using VITEK^®^ 2 Compact (bioMérieux, Marcy-l’Étoile, France) with the appropriate identification (ID) and AST cards containing antibiotic dilutions optimised for the breakpoints defined by the European Committee on Antimicrobial Susceptibility Testing (EUCAST). Specifically, we used GP ID CARD and AST-P659, GN ID CARD, AST-N397 and AST-N439, and YST ID CARD and AST-YS08 to identify and test the susceptibility/resistance patterns of Gram-positive bacteria, Gram-negative bacteria, and yeasts, respectively.

### 2.6. Statistical Analysis

All the data obtained by the surveillance were analysed using Prism 4.0 software. Descriptive statistics were used to calculate mean values, standard deviations, percentage values and Δ variations.

## 3. Results

### 3.1. Overall Samplings

During the 2-year period under consideration, a total of 1122 environmental samplings were carried out. The details are summarised in Table 1.

The table shows the total number of samplings performed, first divided into surface and air samplings. Surface samplings were further divided into those performed in operating rooms and wards. The latter were further divided into routine controls and controls following an alert report. Notably, the number of surface samples collected in wards increased owing to a marked increase in controls conducted following an alert report. Specifically, Table 2 shows the percentage of the alert reports received by our operational unit, divided by notifying ward and presented in descending order.

The table shows that the units most affected by infections caused by alert microorganisms were those caring for particularly susceptible and vulnerable patients. Notably, when the percentage values for individual notifying units were grouped by area, the medical area accounted for the highest proportion of notifications (52.5%), followed by the emergency (29.9%) and surgical (17.6%) areas.

### 3.2. Sampled Surfaces

Overall, 4187 surfaces were tested. The details are summarised in Table 3.

The table shows a marked increase in the number of surfaces tested in wards, especially those sampled during controls following an alert report, in parallel with the increase in sampling described above.

Regarding the positivity rate, no positive results were detected on surfaces in operating rooms. For air samplings, 21.0% (53/252) of samples had non-conforming results, of which 64.1% (34/53) were samples collected “at rest” (these non-conforming samples represented 22.9% (33/144) of all “at rest” air samplings) and 35.9% (19/53) were samples collected in the “operational” way (during the surgical activity) and representing 18.5% (20/108) of the totality of “operational” samples.

Conversely, 732/3057 (23.9%) sampled surfaces from wards tested positive, with very similar positivity rates for the two types of controls. Specifically, 285/1246 (22.9%) surfaces tested during routine controls were positive, compared with 447/1811 (24.7%) during controls following an alert report. However, a temporal difference in positivity rates was observed between the two types of controls. Specifically, for routine controls, positivity decreased from 194/622 (31.2%) surfaces in 2024 to 91/624 (14.6%) in 2025 (Δ% = −53.2%). Conversely, for controls following an alert report, the positivity rate remained stable, with 176/721 (24.4%) surfaces testing positive in 2024 and 271/1090 (24.9%) in 2025 (Δ% = +2.0%).

**Figure 1:**
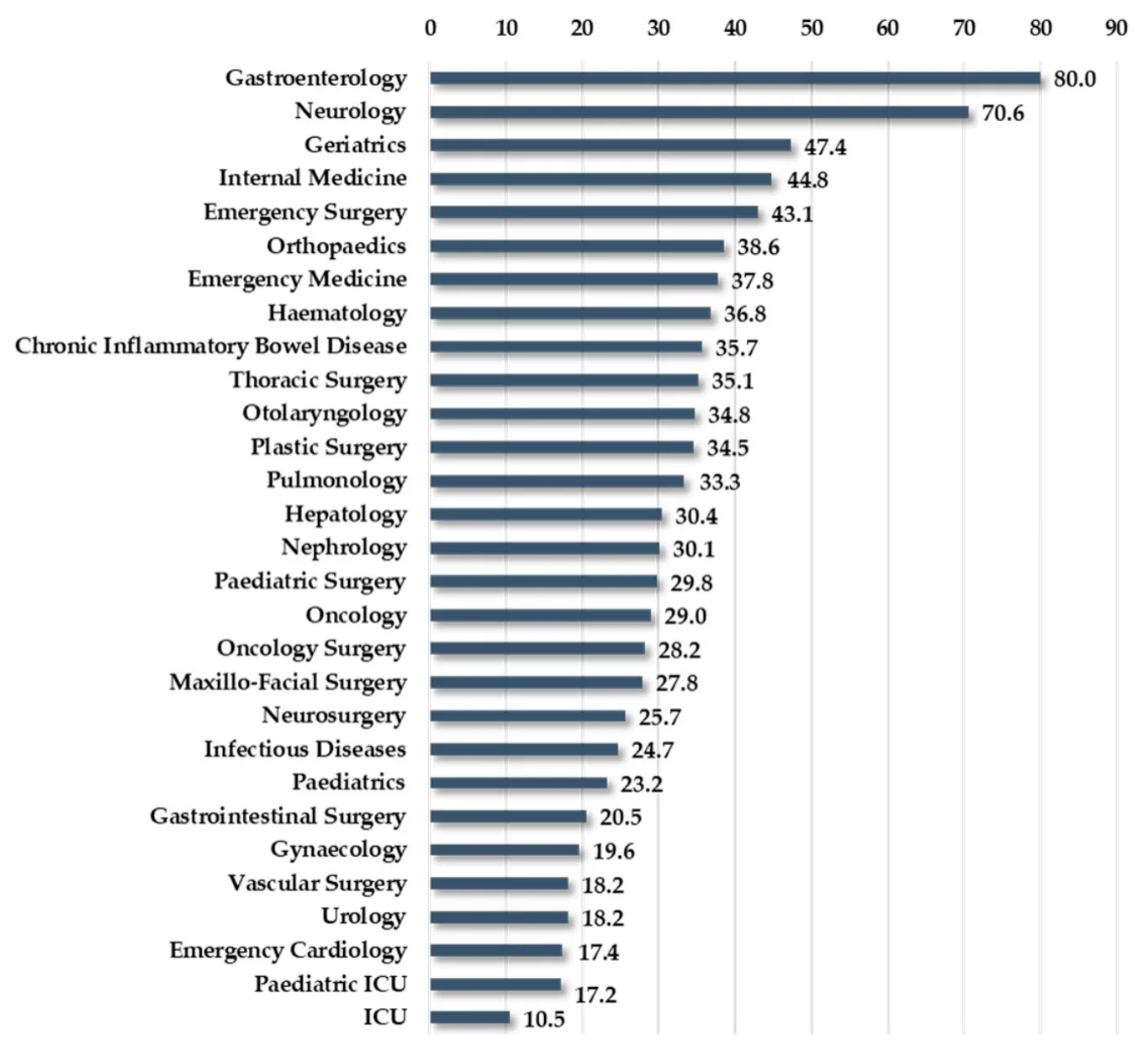
Percentage of positivity of the sampled Operative Units.

We therefore assessed the positivity rate for the various Operative Units, calculated as the number of times they tested positive divided by the number of times they were sampled. The results, in descending order of positivity, are shown in Figure 1.

Figure 1 shows a clearly higher positivity rate in wards belonging to the medical area ($\bar{x}$ = 42.5% ± 17.1), followed by surgical ($\bar{x}$ = 29.6% ± 6.9) and emergency wards ($\bar{x}$ = 27.9% ± 10.2).

**Figure 2:**
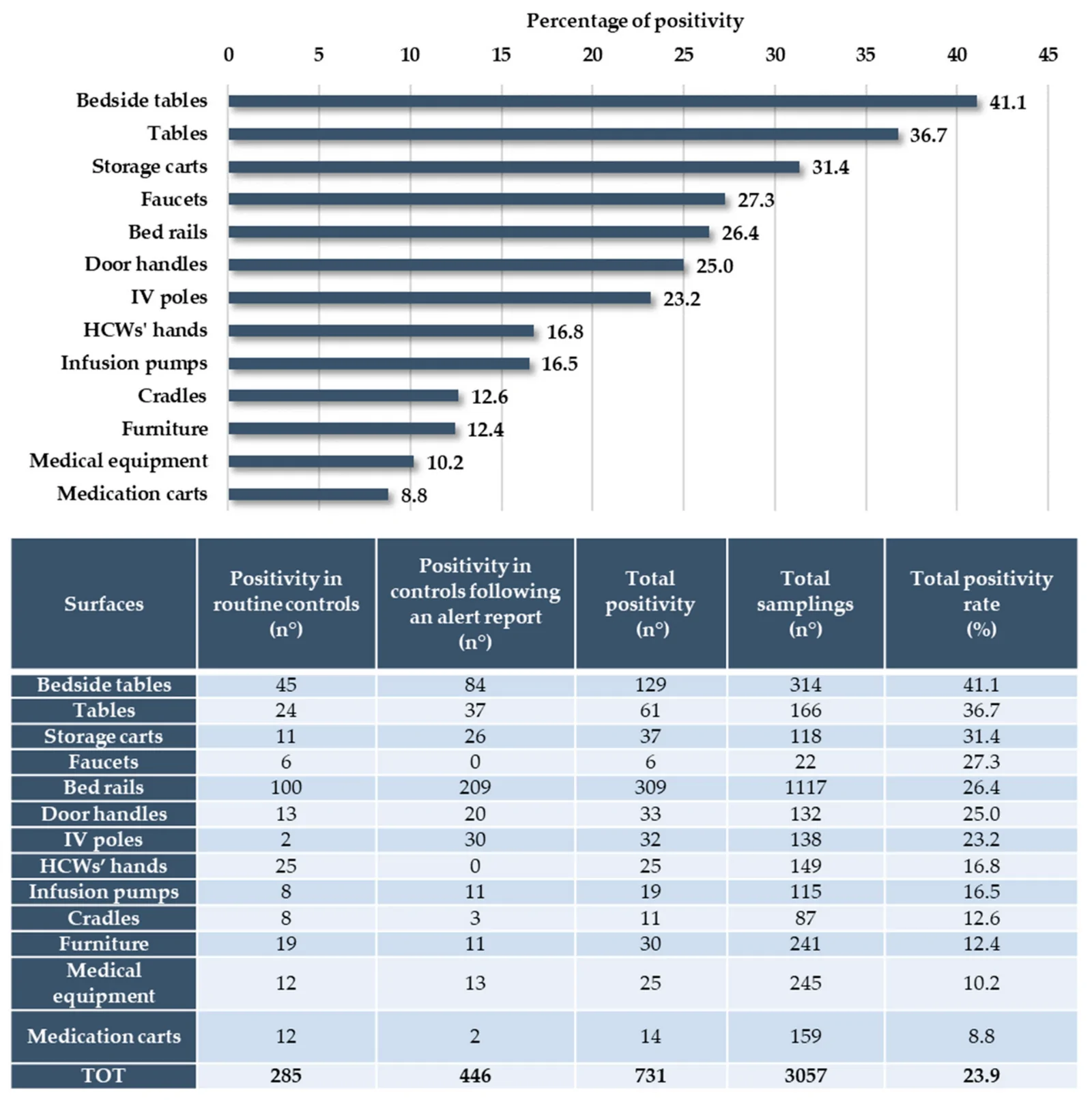
Absolute numbers and percentage values of positivity per surface in two kinds of control: routine and following an alert report.

Likewise, we then calculated the positivity rate for individual surfaces as the number of times they tested positive divided by the number of times they were sampled. The results, in descending order of positivity, are shown in Figure 2.

The figure shows that near-patient surfaces (bed rails and bedside tables) were overall more frequently positive than other surfaces. However, particular attention should be given to some critical high-touch surfaces (IV poles, infusion pumps, cradles, medical equipment, and medication carts), which, despite having lower overall positivity rates, can represent important sources of cross-contamination and infection. Regarding HCWs’ hands, we found a low positivity rate, with coagulase-negative staphylococci (CoNS) being detected most frequently.

### 3.3. Detected Microbial Strains

**Figure 3:**
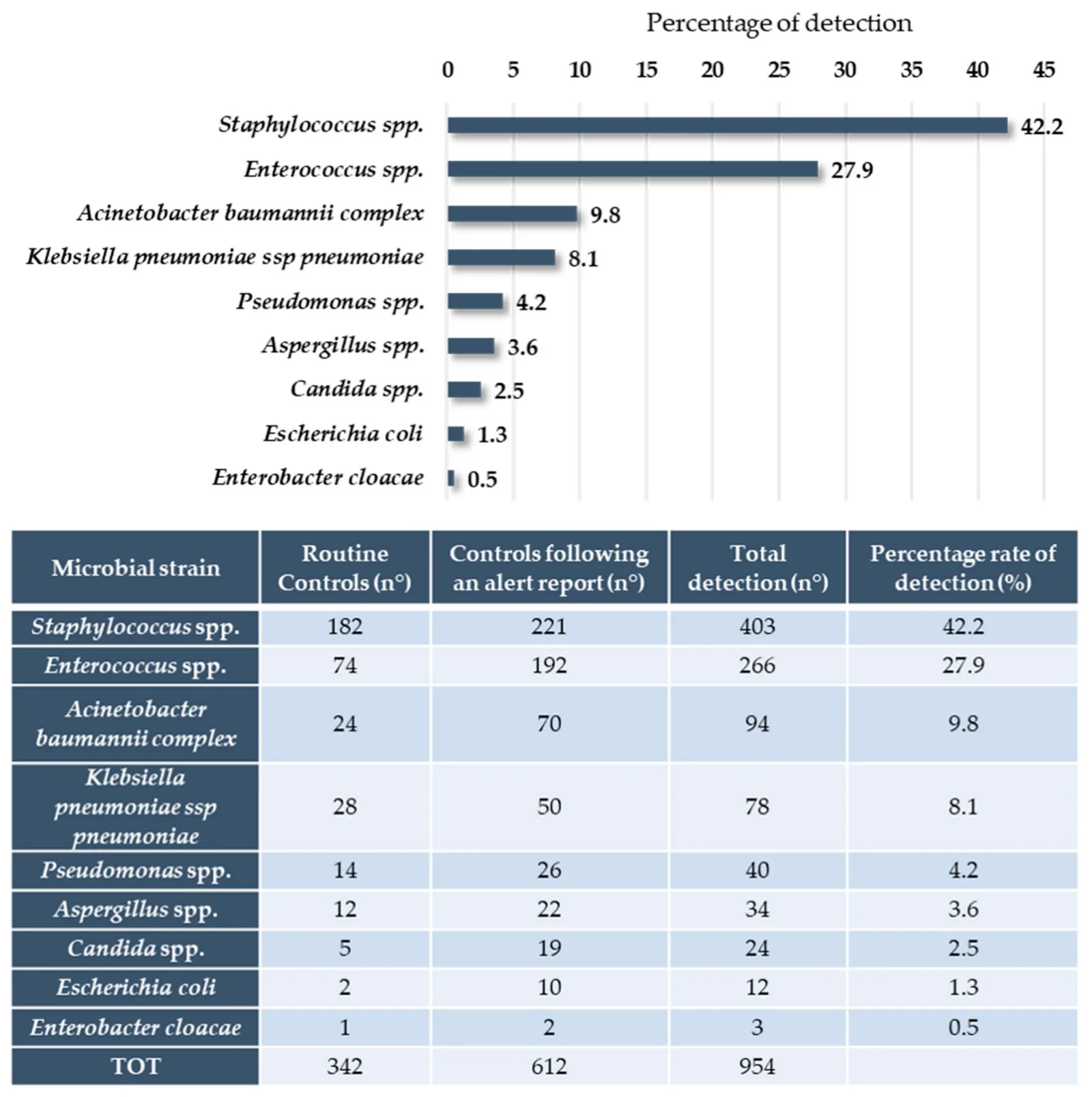
Absolute numbers and percentage rate of detection per single microbial strain.

Overall, 954 microbial strains were detected, with a marked difference among genera and species. Specifically, Gram-positive bacteria accounted for the vast majority of isolates (70.1%, 669/954), followed by Gram-negative bacteria (23.9%, 228/954) and yeasts/moulds (6.0%, 58/954, of which 24 were yeasts and 34 were moulds). Details of the detected isolates are shown in Figure 3.

Among Gram-positive bacteria, *Staphylococcus* spp. accounted for 60.2% (403/669), followed by *Enterococcus* spp. with 39.8% (266/669). Among *Staphylococcus* spp., the detected species, in descending order of detection, were *S. haemolyticus* (48.6%, 196/403), *S. capitis* (14.4%, 58/403), *S. aureus* (8.7%, 35/403), *S. hominis* (8.7%, 35/403), *S. warneri* (8.2%, 33/403), *S. auricularis* (5.5%, 22/403), *S. saprophyticus* (2.9%, 12/403), and *S. cohnii* ssp. *cohnii* (2.9%, 12/403). Among *Enterococcus* spp., the detected species, in descending order of detection, were: *E. faecium* (72.2%, 192/266), *E. faecalis* (22.2%, 59/266) and *E. durans* (5.6%, 15/266).

Regarding Gram-negative bacteria, *Acinetobacter baumannii complex* was the only species detected within the genus *Acinetobacter*. Similarly, *Klebsiella pneumoniae* ssp. *pneumoniae*, *Escherichia coli* and *Enterobacter cloacae* were the only species detected within their respective genera of the *Enterobacteriaceae* family.

Among yeasts, *Candida* spp. was the only genus detected, with *C. albicans* accounting for 58.3% (14/24) followed by *C. parapsilosis* with 41.7% (10/24). Notably, no strain of *Candidozyma auris* was detected. Finally, a remarkable mention has to be made for *Aspergillus* spp, of which *A. niger*, *A. fumigatus* and *A. flavus* accounted for 61.8% (21/34), 23.5% (8/34) and 14.7% (5/34), respectively.

### 3.4. Antimicrobial Resistance Rate

To evaluate the presence and spread of the well-known ESKAPEE pathogens causing HAIs in the hospital environment, we evaluated the resistance/sensitivity patterns of the detected strains and their presence in various wards.

**Figure 4:**
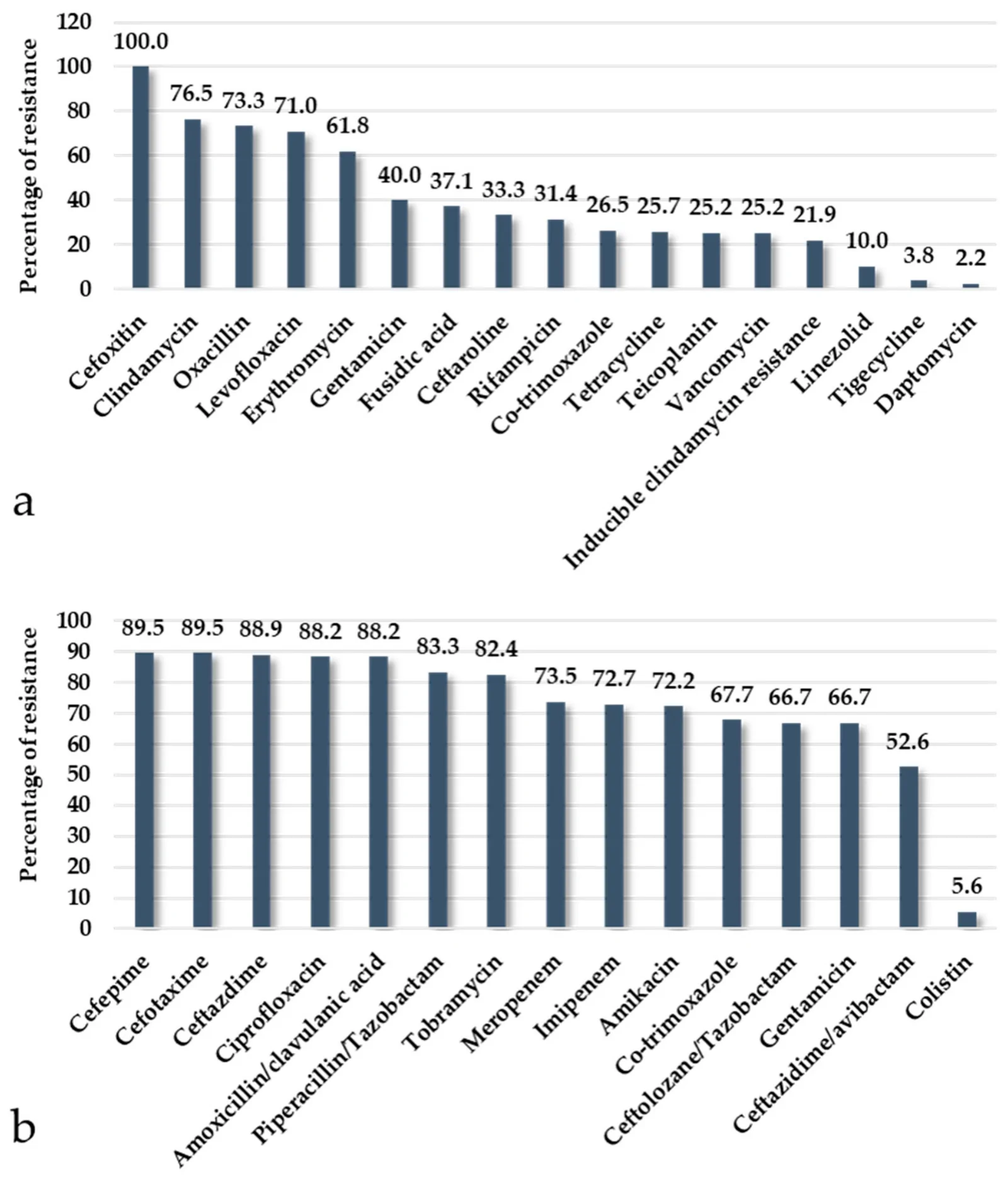
Overall antibiotic resistance in Gram-positive (**a**) and Gram-negative (**b**) detected bacteria.

Figure 4 shows the overall antibiotic resistance detected in Gram-positive and Gram-negative bacteria to the antibiotics tested.

Interestingly, the detected Gram-positive bacteria showed a much lower mean level of resistance, at 39.1% ± 27.0 (min. 2.2%, max 100%), compared with Gram-negative bacteria, which showed a mean level of 72.5% ± 20.9 (min. 5.6%, max 89.5%).

We then evaluated resistance to the different antibiotic classes tested. The results are shown in Table 4.

The table shows high mean levels of resistance (higher than 60%) for the main and broad classes of antibiotics, with some differences among the various members of each class. This was especially evident for cephalosporins, for which the highest levels of resistance were detected for older molecules belonging to the second and third generations, while lower levels were found for molecules combined with beta-lactam inhibitors and for those belonging to the fifth generation. Low levels of resistance were detected for highly specific agents such as glycopeptides, linezolid, colistin, daptomycin and tigecycline, which are used much less frequently on an empirical basis and are therefore less subject to the development of resistance.

Finally, considering individual genera and species, the results are shown in Table 5.

### 3.5. Methicillin-Resistant Staphylococcus aureus (MRSA) and Methicillin-Resistant CoNS (MR-CoNS)

*S. aureus* accounted for only 8.7% of detected staphylococci. However, all the detected strains were methicillin-resistant (100%, 35/35). The strains were detected exclusively on near-patient surfaces such as bed rails and bedside tables. No MRSA was detected on medical equipment or HCWs’ hands. All strains were also resistant to clindamycin and levofloxacin but susceptible to vancomycin (no VISA or VRSA were detected), teicoplanin, and tigecycline. The wards where these strains were detected belonged predominantly to the medical area (80.0%, 28/35) specifically Internal Medicine (40.5%, 11/28), Geriatrics (36.2%, 10/28) and Oncology (23.3%, 7/28). In the surgical area (20%, 7/35), MRSA was isolated in Thoracic Surgery (57.1%, 4/7) and Orthopaedics (42.9%, 3/7). No strains were detected in emergency area wards.

A mention has to be made of CoNS, as they were resistant to methicillin in 53.3% (207/368) of cases. Specifically, 78.6% (154/196) of detected strains of *S. haemolyticus,* 68.6% (24/35) of *S. hominis*, 39.4% (13/33) of *S. warneri* and 27.6% (16/58) of *S. capitis* were methicillin-resistant. No methicillin-resistance was detected in *S. auricularis* and *S. cohnii* ssp *cohnii*. Like MRSA, MR-CoNS were detected predominantly on near-patient surfaces in more than the half of cases [33.3% (69/207) and 28.5% (59/207) in bed rails and bedside tables, respectively]. However, unlike MRSA, a certain amount of MR-CoNS was detected on some “critical” surfaces, such as IV poles (18.8%, 39/207), infusion pumps (11.1%, 23/207) door handles (4.8%, 10/207) and storage carts (2.9%, 6/207). CoNS were also detected on HCWs’ hands, and a small proportion (26.7%) of them were MR-CoNS, specifically *S. haemolyticus* and *S. capitis*. MR-CoNS were detected predominantly in wards in the medical area (47.6%, 99/207), specifically Oncology (58.6%, 58/99), Internal Medicine (14.1%, 14/99), Haematology (14.1%, 14/99) and Neurology (13.1%, 13/99). In the surgical area, the percentage of detection was 28.6% (59/207) and, specifically, the involved wards were General Surgery (50.9%, 30/59), Orthopaedics (25.4%, 15/59), Otolaryngology (15.3%, 9/59) and Neurosurgery (8.5%, 5/59). Finally, 23.8% (49/207) of MR-CoNS were detected in emergency area wards, specifically in Emergency Medicine (71.4%, 35/49), the ICU (20.4%, 10/49) and the paediatric ICU (8.2%, 4/49). It should also be noted that 8.7% (32/368) and 41% (15/368) of the isolates showed resistance to vancomycin and linezolid, respectively.

### 3.6. Vancomycin-Resistant Enterococci (VRE)

VRE accounted for 27.8% (74/266) of all detected enterococci. All strains were VR-*E. faecium*. They were isolated exclusively from near-patient surfaces [bed rails 40.5% (30/74), tables 39.2% (29/74) and bedside tables 20.3% (15/74)]. Regarding wards, 60% (44/74) were detected in medical units [Internal Medicine 47.7% (21/44), Paediatrics 31.8% (14/44) and Haematology 20.5% (9/44)] and 40% (30/74) were detected in emergency units (all in Emergency Medicine). No VRE were isolated from surgical wards. All VRE were susceptible to linezolid.

### 3.7. Multidrug-Resistant (MDR) Acinetobacter baumannii

*Acinetobacter baumannii complex* represented the third most frequently detected microbial strain overall and the most frequently detected Gram-negative species. The most concerning finding regarding this species was its almost complete resistance to all the antibiotics tested. In particular, 100% (94/94) of the isolates were resistant to carbapenems, aminoglycosides, fluoroquinolones and trimethoprim-sulfamethoxazole. However, only 7.7% (7/94) of strains were resistant to colistin. Notably, these strains were detected on near-patient surfaces [bed rails 46.8% (44/94) and bedside tables 26.6% (25/94)] but a substantial number (20.2%, 19/94) were detected on IV poles. Moreover, 6.4% (6/94) were isolated from storage carts. For this strain as well, medical area wards were the most affected (46.6%, 44/94), with Internal Medicine ranking first (45.5%, 20/44), followed by Neurology, Hepatology, Pulmonology and Infectious Diseases, all at 13.7% (6/44). Surgical and emergency wards each accounted for 26.6% (25/94), with Neurosurgery (75.2%, 19/25) and Oncology Surgery (24.8%, 6/25) in the surgical area and Emergency Medicine (75.2%, 19/25) and the ICU (24.8%, 6/25) for the emergency area.

### 3.8. Carbapenem-Resistant Enterobacteriaceae (CRE)

CRE were detected in different wards and on different surfaces. In particular, all CRE strains were *Klebsiella pneumoniae* ssp. *pneumoniae*, as neither the detected *Escherichia coli* nor *Enterobacter cloacae* were resistant to carbapenems. Specifically, 66.7% (52/78) of the detected *K. pneumoniae* strains were resistant to carbapenems. These strains were isolated from near-patient surfaces [75.0% (39/52) bed rails and 17.3% (9/52) bedside tables], while 7.7% (4/52) were detected on IV poles. Moreover, the most affected wards were those belonging to the medical area (75.0%, 39/52), followed by emergency wards (17.3%, 9/52) and surgical wards (7.7%, 4/52). Specifically, in the medical area, the wards involved were Geriatrics (33.3%, 13/39), Oncology (23.1%, 9/39), Internal Medicine (12.8%, 5/39), Neurology (10.3%, 4/39), Hepatology (10.2%, 4/39) and Infectious Diseases (10.3%, 4/39). The emergency wards involved were Emergency Medicine (66.7%, 6/9) and the ICU (33.3%, 3/9). Finally, in the surgical area, 75.0% (3/4) of strains were detected in Neurosurgery and 25.0% (1/4) in General Surgery.

### 3.9. MDR-Pseudomonas aeruginosa

*Pseudomonas aeruginosa* represented only a small proportion (17.5%, 7/40) of all species belonging to the genus *Pseudomonas* spp. The other species detected were *P. putida* (32.5%, 13/40), *P. fluorescens* (22.5%, 9/40), *P. stutzeri* (15.0%, 6/40) and *P. oryzihabitans* (12.5%, 5/40). These bacteria were predominantly on taps (42.5%, 17/40) but also on some near-patient surfaces, such as bed rails (25.0%, 10/40), bedside tables (20.0%, 8/40) and tables (12.5%, 5/40). A very small proportion (5.0%, 2/40) was detected on HCWs’ hands, with *P. aeruginosa* accounting for 100% of these isolates. *P. aeruginosa* strains showed substantial levels of antibiotic resistance, with 71.4% (5/7) resistant to piperacillin-tazobactam and 42.9% (3/7) resistant to carbapenems. Conversely, the other detected species did not show an important level of antibiotic resistance and were susceptible to most of the antibiotics tested.

### 3.10. Candida spp.

*Candida* spp. was the only genus of yeasts detected. Bed rails (41.7%, 10/24) and tables (25.0%, 6/24) were the most common surfaces from which these microorganisms were isolated, but some isolates were also recovered from medication carts (16.7%, 4/24) and medical equipment (16.7%, 4/24). Regarding antifungal resistance, only 20.8% (5/24) of strains were resistant to fluconazole, while no resistance to voriconazole, caspofungin and micafungin was detected. The resistant strains were detected on bedrails in the ICU and Emergency Medicine.

## 4. Discussion

A large body of scientific evidence has demonstrated the role of the contaminated hospital environment as a source of dangerous pathogens causing HAIs [28,29]. In this setting, complex mechanisms of cross-contamination occur between inanimate surfaces, HCWs, and patients. In this dangerous game, HCWs’ hands undoubtedly play a crucial role, becoming contaminated and, in turn, contaminating patients, surfaces and medical devices [30]. For this reason, hand hygiene and environmental sanitation in healthcare facilities are considered the cornerstones of HAI prevention and the most cost-effective interventions [31]. However, the potential role of microbiological monitoring of the hospital environment in HAI prevention is still not universally recognised.

This study examined how monitoring microbiological contamination of the hospital environment could play a supplementary role in helping to combat HAIs, by providing a plausible picture of the actual level of microbiological contamination, including ESKAPEE microorganisms, within a large acute-care hospital. Defining the environmental distribution and resistance landscape of such organisms within the hospital could provide indirect evidence of the need to implement environmental sanitation protocols and, above all, help identify areas of concern that may require action.

In our setting, surgical rooms consistently showed an absence of microorganisms on the surfaces tested, reflecting the great care taken with these particularly critical environments. However, this does not mean that operating rooms are completely safe, given the inherent limitations of environmental sampling, such as random and occasional surface testing. Unlike surface samples, a certain percentage of air samples had non-conforming results. This finding could probably be closely related to a failure to maintain the ventilation system properly and some poor practices that we encountered during our checks in the operating rooms, such as keeping the doors open during surgical procedures, thereby impairing the filtering function of the HEPA system within the rooms, and overcrowding because of an occasionally excessive number of team members and, above all, students. This result must be carefully considered, given that air quality without microbiological contamination has been shown to be essential in preventing SSIs [32].

A completely different approach is required for patient care areas, which were often found to be heavily and dangerously contaminated. A particularly concerning result was the marked increase in alert notification occurring in the last year, which may be an indirect sign of the high incidence of HAIs and potential cross-contamination due to environmental circulation of ESKAPEE pathogens. This increase could be due to a decline in the adoption of well-known best practices that are effective in reducing HAIs. To address this significant issue, our healthcare facility has launched a programme of training events aimed at all professionals involved in the HAI prevention, during which, amongst other information, we presented our findings on hospital microbiological monitoring. We believe, in fact, that one potential use of such research is to serve as a basis for raising awareness amongst healthcare professionals about the importance of adopting appropriate preventive measures. As emphasised by previous evidence, health education is one of the cornerstones that a healthcare facility can use to improve HCWs’ awareness of HAIs [33,34]. This research can therefore serve as a valuable tool for health education and the promotion of a safety culture, helping to enhance knowledge and, above all, raise awareness that ESKAPEE microorganisms can be found in large numbers in the hospital environment and can therefore be easily transmitted to patients during routine care activities if the appropriate precautions are not taken.

One particularly important finding was that the average positivity rate remained fairly constant over the 2 years. However, the difference in positivity rates between the two types of control (showing a sharp decline for routine controls and, conversely, an increase for controls following an alert) demonstrates how infected patients can contaminate their immediate surroundings [35]. Indeed, one of the main safeguards in HAI prevention is the physical or, at the very least, functional isolation of infected patients, with possible cohorting and the correct application of contact precautions [36]. During our control activities, we observed that these recommendations were often not fully followed, and this may explain the increase in the positivity rate reported in 2025, which coincided with the marked increase in alert notifications. An infected or contaminated patient cared for without appropriate contact precautions can easily contaminate the hospital environment. This gap was particularly evident in wards belonging to the medical area, such as Internal Medicine, Gastroenterology, Geriatrics, and others, where we detected higher overall positivity rates. This finding may be due to particular conditions, among which, overcrowding could play a leading role. These wards are often crowded with not only inpatients and healthcare professionals but also relatives and medical and nursing students, as our facility is a university hospital. Besides being a source of microorganisms, overcrowding in a medical ward can lead to failures to adhere to basic best practices, potentially resulting in inadequate cleaning, contaminated shared equipment, and breakdowns in hand hygiene [37,38]. Moreover, we believe that an excessive number of inpatients constantly present in medical wards may exacerbate chronic staff shortages, both among healthcare staff—resulting in a lower HCW-to-patient ratio—and among staff dedicated to environmental sanitisation, which may be a critical factor in the development of HAIs [39,40].

Regarding the positivity rate detected on surfaces, our results are in line with international scientific evidence showing that near-patient and high-touch surfaces, such as bed rails and bedside tables, are particularly likely to be contaminated and can therefore serve as sources of infection through cross-contamination, transferring microorganisms to HCWs’ hands or directly to patients and driving the spread of HAIs [41,42]. At the same time, some “critical” items such as medication carts, IV poles, infusion pumps, and medical equipment tested positive in a certain proportion of cases. These surfaces may pose a greater risk of cross-contamination than others, as they are frequently touched by healthcare staff [43,44,45].

Overall, a wide variety of microorganisms were detected on the various surfaces sampled. Of these, Gram-positive species such as staphylococci and enterococci were by far the most prevalent. These microorganisms frequently colonise human skin and are therefore indicative of human-associated contamination of the environment. One particularly interesting finding was the low prevalence of *S. aureus* compared with CoNS. This may be because CoNS constitute the vast majority of normal human skin flora and are continuously shed onto hospital surfaces, making them ubiquitous throughout clinical environments. This finding is in line with previous evidence showing that CoNS are by far the most detected species on hospital environmental surfaces, owing in part to their ability to form resistant biofilm, and can be transferred to patients, causing severe infections [46,47,48]. Among Gram-negative species, the well-known *A. baumannii complex*, *K. pneumoniae* ssp. *pneumoniae* and *Pseudomonas* spp. were the most frequently detected, confirming the well-known ability of these microorganisms to spread, colonise, and persist on hospital surfaces [49,50,51]. Finally, fungal pathogens should also be noted; we detected yeasts of the genus *Candida* and moulds of the genus *Aspergillus*. Fungi are widespread in hospital environments and can cause important HAIs, especially in particularly fragile patients [52,53].

One of the most important aims of such research was to analyse the spread of antimicrobial-resistant strains within the hospital environment in order to assess the risk to which patients are exposed. In this regard, it is important to consider the significant level of antibiotic resistance found in Gram-negative species, which were significantly more resistant than Gram-positive strains. This finding is consistent with the scientific evidence, as it is well-known that Gram-negative bacteria are more capable of developing resistance to antibiotics than Gram-positive bacteria because of their structural characteristics and genetic plasticity [54,55]. Regarding resistance to the antibiotic classes tested, our findings were fairly predictable, given the widespread use in hospitals—particularly for empirical treatment—of the main classes of antibiotics and, within each class, of the compounds that have been in use for the longest time (for example, amoxicillin-clavulanic acid for penicillins, or first- and second-generation compounds for cephalosporins) [56].

Among Gram-positive bacteria, our attention focused on methicillin-resistant strains of staphylococci and vancomycin-resistant strains of enterococci. Over the years, staphylococci have become one of the most common groups of microorganisms responsible for HAIs at different body sites and have, over time, developed a significant degree of resistance to antibiotics [57,58,59]. Furthermore, they are capable of forming biofilms on surfaces and medical devices, thereby enabling them to survive for long periods in the hospital environment [60]. It should be emphasised that these strains have shown a significant level of resistance to anti-MRSA antibiotics such as vancomycin and linezolid, a finding in line with previous evidence [61,62]. Widespread contamination of the hospital environment by MDR-CoNS is considered a concern not only because these bacteria are able to form biofilms and cause HAIs but also because of their ability to act as reservoirs of resistance genes that can be transferred to other bacteria, such as *S. aureus* and enterococci [63,64].

Among enterococci, the most critical concern is the acquisition of vancomycin resistance, especially by *E. faecium*, which is one of the most important ESKAPEE pathogens [65,66]. Studies have shown the spread of these microorganisms within hospitals and their ability to survive on surfaces and devices for long periods. VRE have been shown to survive on dry inanimate surfaces for 5 days to more than 4 months and on certain fabrics and medical equipment for up to 3 years [67,68]. In our setting, VRE represented about one-third of all the resistant entericocci and were detected especially on near-patient surfaces, again indicating the key role played by contaminated/infected patients in spreading pathogens. Their detection in the environment of emergency wards is a particularly critical issue, given the high susceptibility of inpatients to developing HAIs in these settings.

Among Gram-negative species, *A. baumannii complex* was confirmed as the most prevalent and antibiotic-resistant Gram-negative environmental bacterial species, in line with the international literature [49,69,70]. Another significant finding was *K. pneumoniae* ssp. *pneumoniae*, which is now a major cause of infections caused by CRE [71]. The most significant concerns for both these bacteria were their high resistance to the antibiotics tested and, above all, the surfaces and wards where they were detected. It is worth emphasising their presence on particularly critical surfaces such as IV poles and medication carts, from which they can easily contaminate HCWs’ hands and, consequently, patients. Furthermore, their presence in high-risk wards, particularly ICUs, makes them a serious threat. Both these ESKAPEE pathogens can survive for long periods on dry hospital surfaces. In particular, *A. baumannii complex* has been shown to have a particularly long survival time in the hospital environment [7].

In light of the above, it is clear that the hospital environment can be extensively and dangerously contaminated by ESKAPEE pathogens; for this reason, it is essential to rethink and emphasise the role of environmental sanitation processes in HAI prevention, as indicated by international evidence [72,73]. However, it has recently been highlighted that microbial resistance to disinfectants is spreading globally, and this could represent a new challenge in HAI prevention [74,75]. One possible solution could be the use of alternative disinfectant systems, such as probiotic-based systems, as a possible alternative to standard chemical disinfection. In fact, probiotic-based sanitation systems have been shown to reduce pathogenic colonisation on hospital surfaces by up to 90%. By using eco-friendly detergents containing *Bacillus* spores, these biological cleaners suppress harmful microbes through resource competition and the release of antimicrobial metabolites while preventing the emergence of drug-resistant strains [76,77]. Another particularly interesting area of research is the study of innovative materials with intrinsic antibacterial and anti-biofilm properties for use in the manufacture of surfaces and, above all, medical devices. Such materials would be capable of preventing microbial adhesion and, consequently, the formation of dangerous biofilms, thereby making medical devices resistant to contamination by pathogenic species [78,79,80,81].

## 5. Conclusions

Microbiological monitoring of the hospital environment proves to be a valuable source of information regarding the characterisation of hospital microbiota and, especially, the presence and circulation of ESKAPEE pathogens within the hospital, making it possible to identify critical areas within wards and the surfaces that are most heavily contaminated and, therefore, pose the greatest risk to vulnerable patients. This practice also helps to identify where action is needed by enhancing sanitisation procedures and, above all, encouraging appropriate behaviour by healthcare staff. For these reasons, this practice could complement the existing prevention procedures in various ways, adding a further element to the ongoing fight against AMR and HAIs.

## 6. Limitations of the Study

This research has some limitations that have to be considered. Firstly, it was a descriptive, single-centre, retrospective study with a mix of routine and alert-triggered investigations. Furthermore, environmental microbiological tests were performed randomly in limited areas of the environment, providing only a partial picture of the actual level of environmental contamination. In addition, culture methods can inherently show some heterogeneity depending on the expertise and experience of the person taking the sample and the person analysing it in the laboratory. Finally, the lack of molecular comparison with clinical isolates and the absence of patient HAI outcomes limit the generalisability of the research.

## Figures and Tables

**Table 1:** Summary of samplings performed in the two-year period under study.

**OVERALL SURFACE SAMPLINGS**			
TOTAL 870 (77.5%)	2024 405 (46.5%)	2025 465 (53.5%)	Δ% +14.8%
**Samplings in Operating Rooms**			
TOTAL 373 (42.9%)	2024 192 (51.5%)	2025 181 (48.5%)	Δ% −5.7%
**Samplings in Wards**			
TOTAL 497 (57.1%)	2024 213 (42.9%)	2025 284 (57.1%)	Δ% +33.3%
***Routine controls in wards***			
TOTAL 116 (23.3%)	2024 64 (55.2%)	2025 52 (44.8%)	Δ% −18.7%
***Controls in wards following alert report***			
TOTAL 381 (76.7%)	2024 149 (39.1%)	2025 232 (60.1%)	Δ% +55.7%
**OVERALL AIR SAMPLINGS**			
TOTAL 252 (22.5%)	2024 132 (52.4%)	2025 120 (47.6%)	Δ% −9.1%
**“At rest”**			
TOTAL 144 (57.1)	2024 72 (50.0%)	2025 72 (50.0%)	Δ% 0%
**“Operational”**			
TOTAL 108 (42.9%)	2024 58 (53.7%)	2025 50 (46.3%)	Δ% −13.8%

**Table 2:** Percentage of alert reports received by the Hospital Hygiene Operative Unit in the period under study.

OPERATIVE UNITS	PERCENTAGE OF ALERT REPORTS
Intensive Care Unit (ICU)	17.0%
Oncology	10.6%
Emergency Medicine	10.0%
Internal Medicine	7.3%
Neurosurgery	6.5%
Infectious Diseases	5.6%
Liver Diseases	5.0%
Paediatrics	4.7%
Haematology	3.5%
Neurology	2.6%
Neonatal Intensive Care Unit	2.6%
Nephrology	2.6%
Pulmonology	2.3%
Oncology Surgery	2.3%
Geriatrics	2.3%
Gastrointestinal Surgery	2.1%
Vascular Surgery	2.1%
Paediatric Gastroenterology	1.8%
Cardiology	1.8%
Inflammatory Bowel Disease	1.8%
Paediatric Surgery	0.9%
Urology	0.9%
Emergency Surgery	0.6%
Orthopaedics	0.6%
Gastroenterology	0.3%
Thoracic Surgery	0.3%
Gynaecology	0.3%
Allergic Diseases	0.3%
Maxillofacial Surgery	0.3%
Plastic Surgery	0.3%
Paediatric Emergency	0.3%
General Surgery	0.3%
Otolaryngology	0.3%

**Table 3:** Summary of surfaces sampled in the two-year period under study.

OVERALL SURFACES SAMPLED			
TOTAL 4187	2024 1934 (46.2%)	2025 2253 (53.8%)	Δ% +16.5%
**Samplings in Operating Rooms**			
TOTAL 1130 (27.0%)	2024 591 (52.3%)	2025 539 (47.7%)	Δ% −8.8%
**Samplings in Wards**			
TOTAL 3057 (73.0%)	2024 1343 (43.9%)	2025 1714 (56.1%)	Δ% +27.6%
***Routine controls in wards***			
TOTAL 1246 (40.8%)	2024 622 (49.9%)	2025 624 (50.1%)	Δ% +0.3%
***Controls in wards following alert report***			
TOTAL 1811 (59.2%)	2024 721 (39.8%)	2025 1090 (60.2%)	Δ% +51.2%

**Table 4:** Overall percentage of resistance shown by detected microbial isolates towards tested antibiotics, grouped by class and in descending order of resistance.

**PENICILLINS 81.6% ± 6.2** Amoxicillin-Clavulanic Acid 88.2% Piperacillin/Tazobactam 83.3% Oxacillin 73.3%	**MACROLIDES 61.8%** Erythromycin 61.8%
**FLUOROQUINOLONES 79.6% ± 8.6** Ciprofloxacin 88.2% Levofloxacin 71.0%	**CO-TRIMOXAZOLE 46.6%**
**LINCOSAMIDES 76.5%** Clindamycin 76.5%	
**CEPHALOSPORINS 74.4% ± 22.5** Cefoxitin 100% Cefotaxime 89.5% Cefepime 89.5% Ceftazidime 88.9% Ceftolozane/Tazobactam 66.7% Ceftazidime/Avibactam 52.6% Ceftaroline 33.3%	
	**FUSIDIC ACID 37.1%**
	**RIFAMPICIN 31.4%**
	**TETRACYCLINE 25.7%**
	**GLYCOPEPTIDES 25.2%** Teicoplanin 25.2% Vancomycin 25.2%
**CARBAPENEMS 73.1 ± 0.4** Meropenem 73.5% Imipenem 72.7%	**OXAZOLIDINONES 10.0%** Linezolid 10.0%
	**LIPOPEPTIDES 3.9% ± 1.7** Colistin 5.6% Daptomycin 2.2%
**AMINOGLYCOSIDES 69.3% ± 15.7** Tobramycin 82.4% Amikacin 72.2% Gentamicin 53.3%	
	**TIGECYCLINE 3.8%**

**Table 5:** Percentage of resistance (absolute numbers) divided by detected strains and tested antimicrobials.

**GRAM-POSITIVE BACTERIA**																	
	**FOX**	**OXA**	**CFT**	**GEN**	**LEV**	**DA**	**ERY**	**LZD**	**DAP**	**TEC**	**VAN**	**TET**	**TGC**	**FA**	**RIF**	**SXT**	$\bar{x}$
*S. aureus*	100% (35/35)	100% (35/35)	34.3% (12/35)	31.4 (11/35)	65.7% (23/35)	65.7% (23/35)	34.3% (12/35)	0.0% (0/35)	0.0% (0/35)	0.0% (0/35)	0.0% (0/35)	31.4 (11/35)	0.0% (0/35)	60.0% (21/35)	34.3 (12/35)	28.6 (10/35)	36.6%
CoNS	75.3% (277/368)	56.2% (207/368)	0.0% (0/368)	40.5% (149/368)	100% (368/368)	76.6 (282/368)	63.3 (233/368)	4.1% (15/368)	0.0% (0/368)	8.7% (32/368)	8.7% (32/368)	25.0 (92/368)	0.0% (0/368)	32.3% (119/368)	29.1 (107/368)	25.8% (95/368)	34.1%
*Enterococcus* spp.	//	//	//	//	50.0% (133/266)	//	//	0.0% (0/266)	//	27.8% (74/266)	27.8% (74/266)	//	11.6% (31/266)	//	//	//	23.4%
**GRAM-NEGATIVE BACTERIA**																	
	**AMC**	**TZP**	**CTX**	**CAZ**	**CZA**	**CT**	**CEF**	**IPM**	**MER**	**AMK**	**GEN**	**TIP**	**CYP**	**CS**	**SXT**	**//**	$\bar{x}$
*A. baumannii complex*	//	//	//	//	//	//	//	100% (94/94)	100% (94/94)	100% (94/94)	100% (94/94)	100% (94/94)	100% (94/94)	7.7% (7/94)	100% (94/94)		88.5%
*K. pneumoniae* ssp. *pneumoniae*	87.2% (68/78)	87.2% (68/78)	88.5% (69/78)	87.2% (68/78)	56.4% (44/78)	71.8% (56/78)	88.5% (69/78)	66.7% (52/78)	66.7% (52/78)	61.5% (48/78)	38.5% (30/78)	78.2% (61/78)	78.2% (61/78)	0.0 (0/78)	44.9% (35/78)		66.8%
*Pseudomonas* spp.	//	32.5% (13/40)	//	32.5% (13/40)	17.5% (7/40)	12.5% (5/40)	30% (12/40)	7.5% (3/40)	7.5% (3/40)	25.0% (10/40)	25.0% (10/40)	22.5% (9/40)	17.5% (7/40)	0.0% (0/40)	//		19.2%
*E. coli*	41.7% (5/12)	8.3 (1/12)	33.3% (4/12)	25,0% (3/12)	0.0% (0/12)	8.3 (1/12)	25.0% (3/12)	0.0% (0/12)	0.0% (0/12)	8.3 (1/12)	16.7% (2/12)	16.7% (2/12)	41.7% (5/12)	0.0% (0/12)	33.3% (4/12)		17.2%
*E. cloacae*	100.0% (3/3)	66.7% (2/3)	66.7% (2/3)	66.7% (2/3)	66.7% (2/3)	66.7% (2/3)	66.7% (2/3)	0.0% (0/3)	0.0% (0/3)	33.3 (1/3)	66.7% (2/3)	66.7% (2/3)	33.3 (1/3)	0.0 (0/3)	//		59.5%
**YEASTS**																	
	**FCZ**	**VRC**	**CAS**	**MFG**	$\bar{x}$												
*C. albicans*	35.7% (5/14)	0.0% (0/14)	0.0% (0/14)	0.0% (0/14)	8.9%												
*C. parapsilosis*	0.0% (0/10)	0.0% (0/10)	0.0% (0/10)	0.0% (0/10)	0.0%												

**Legend**: FOX = cefoxitin, OXA = oxacillin, CFT = ceftaroline, GEN = gentamicin, LEV = levofloxacin, DA = clindamycin, ERY = erithromycin, LZD = linezolid, DAP = daptomycin, TEC = tecoplanin, VAN = vancomycin, TET = tetracycline, TGC = tigecycline, FA = fusidic acid, RIF = rifampicin, SXT = co-trimoxazole, AMC = amoxicillin-clavulanic acid, TZP = piperacillin-tazobactam, CTX = cefotaxime, CAZ = ceftazidime, CZA = ceftazidime-avibactam, CT = ceftolozane-tazobactam, CEF = cefepime, IPM = imipenem, MER = meropenem, AMK = amikacin, TIP = tobramycin, CYP = ciprofloxacin, CS = colistin, FCZ = fluconazole, VRC = voriconazole, CAS = caspofungin, MFG = micafungin, $\bar{x}$ = mean value, // = no data.

## Data Availability

The raw data supporting the conclusions of this article will be made available by the authors on request.

## References

[1] Chemaly R.F., Simmons S., Dale C., Ghantoji S.S., Rodriguez M., Gubb J., Stachowiak J., Stibich M. (2014). The role of the healthcare environment in the spread of multidrug-resistant organisms: Update on current best practices for containment. Ther. Adv. Infect. Dis..

[2] Cruz-López F., Martínez-Meléndez A., Garza-González E. (2023). How Does Hospital Microbiota Contribute to Healthcare-Associated Infections?. Microorganisms.

[3] Khan M.T., Ribeiro-Almeida M., Yaman U., Prata J.C. (2025). Healthcare Facilities as an Emerging Source of Antimicrobial Resistance: A One Health Perspective. Environments.

[4] World Health Organization (2017). WHO Publishes List of Bacteria for Which New Antibiotics Are Urgently Needed. https://www.who.int/news/item/27-02-2017-who-publishes-list-of-bacteria-for-which-new-antibiotics-are-urgently-needed#:~:text=The%20World%20Health%20Organization%20(WHO)%20published%20a,devices%20such%20as%20ventilators%20and%20blood%20catheters.

[5] Santajit S., Indrawattana N. (2016). Mechanisms of Antimicrobial Resistance in ESKAPE Pathogens. Biomed. Res. Int..

[6] Facciolà A., Pellicanò G.F., Visalli G., Paolucci I.A., Venanzi Rullo E., Ceccarelli M., D’Aleo F., Di Pietro A., Squeri R., Nunnari G. (2019). The role of the hospital environment in the healthcare-associated infections: A general review of the literature. Eur. Rev. Med. Pharmacol. Sci..

[7] Katzenberger R.H., Rösel A., Vonberg R.P. (2021). Bacterial survival on inanimate surfaces: A field study. BMC Res. Notes.

[8] Porter L., Sultan O., Mitchell B.G., Jenney A., Kiernan M., Brewster D.J., Russo P.L. (2024). How long do nosocomial pathogens persist on inanimate surfaces? A scoping review. J. Hosp. Infect..

[9] Garvey M. (2023). Medical Device-Associated Healthcare Infections: Sterilization and the Potential of Novel Biological Approaches to Ensure Patient Safety. Int. J. Mol. Sci..

[10] La Fauci V., Riso R., Facciolà A., Merlina V., Squeri R. (2016). Surveillance of microbiological contamination and correct use of protective lead garments. Annali di Igiene.

[11] La Fauci V., Costa G.B., Facciolà A., Conti A., Riso R., Squeri R. (2017). Humidifiers for oxygen therapy: What risk for reusable and disposable devices?. J. Prev. Med. Hyg..

[12] Akinbobola A.B., Osunla A.C., Bello O.M., Ajayi O.A. (2022). Study of the persistence of selected Gram-negative bacteria pathogens of healthcare-associated infections on hospital fabrics. Am. J. Infect. Control..

[13] Dhayhi N., Kameli N., Salawi M., Shajri A., Basode V.K., Algaissi A., Alamer E., Darraj M., Shrwani K., Alhazmi A.H. (2023). Bacterial Contamination of Mobile Phones Used by Healthcare Workers in Critical Care Units: A Cross-Sectional Study from Saudi Arabia. Microorganisms.

[14] Jinadatha C., Navarathna T., Negron-Diaz J., Ghamande G., Corona B.A., Adrianza A., Coppin J.D., Choi H., Chatterjee P. (2024). Understanding the significance of microbiota recovered from health care surfaces. Am. J. Infect. Control.

[15] Center for Disease Control and Prevention (2024). About Hand Hygiene for Patients in Healthcare Settings. https://www.cdc.gov/clean-hands/about/hand-hygiene-for-healthcare.html.

[16] Peters A., Schmid M.N., Parneix P., Lebowitz D., de Kraker M., Sauser J., Zingg W., Pittet D. (2022). Impact of environmental hygiene interventions on healthcare-associated infections and patient colonization: A systematic review. Antimicrob. Resist. Infect. Control.

[17] Li R., Wang Z., Huang M., Liao D., Yuan Z., Qian K. (2025). Environmental hygiene and healthcare-associated infection: A time-series study based on generalized additive model. Front. Public Health.

[18] Center for Disease Control and Prevention (2024). Standard Precautions for All Patient Care. https://www.cdc.gov/infection-control/hcp/basics/standard-precautions.html.

[19] Center for Disease Control and Prevention (2023). Factors Affecting the Efficacy of Disinfection and Sterilization. https://www.cdc.gov/infection-control/hcp/disinfection-sterilization/efficacy-factors.html.

[20] Jukola S., Gadebusch Bondio M. (2023). Not in their hands only: Hospital hygiene, evidence and collective moral responsibility. Med. Health Care Philos..

[21] Zeinali A., Platt L.S. (2025). Surface materials and cleaning efficacy in healthcare: A comprehensive review of strategies and outcomes. Build. Environ..

[22] Ziegler M.J., Babcock H.H., Welbel S.F., Warren D.K., Trick W.E., Tolomeo P., Omorogbe J., Garcia D., Habrock-Bach T., Donceras O. (2022). Stopping Hospital Infections With Environmental Services (SHINE): A Cluster-randomized Trial of Intensive Monitoring Methods for Terminal Room Cleaning on Rates of Multidrug-resistant Organisms in the Intensive Care Unit. Clin. Infect. Dis..

[23] Gastaldi S., Accorgi D., D’Ancona F. (2025). Tools and strategies for monitoring hospital environmental hygiene services. J. Hosp. Infect..

[24] Santella B., Donato A., Fortino L., Satriani V., Ferrara R.F., Santoro E., Longanella W., Franci G., Capunzo M., Boccia G. (2025). Clean to Prevent, Monitor to Protect: A Scoping Review on Strategies for Monitoring Cleaning in Hospitals to Prevent HAIs. Infect. Dis. Rep..

[25] Assadian O., Harbarth S., Vos M., Knobloch J.K., Asensio A., Widmer A.F. (2021). Practical recommendations for routine cleaning and disinfection procedures in healthcare institutions: A narrative review. J. Hosp. Infect..

[26] World Health Organization (2024). WHO Bacterial Priority Pathogens List, 2024.

[27] Istituto Superiore per la Prevenzione e la Sicurezza del Lavoro (INAIL) (2009). Linee Guida Sugli Standard di Sicurezza e di Igiene del Lavoro nel Reparto Operatorio. https://www.ospedalesicuro.eu/attachments/article/144/ISPESL-LG-SaleOperatorie.pdf.

[28] Otter J.A., Yezli S., Salkeld J.A., French G.L. (2013). Evidence that contaminated surfaces contribute to the transmission of hospital pathogens and an overview of strategies to address contaminated surfaces in hospital settings. Am. J. Infect. Control.

[29] Weber D.J., Anderson D., Rutala W.A. (2013). The role of the surface environment in healthcare-associated infections. Curr. Opin. Infect. Dis..

[30] Mouajou V., Adams K., DeLisle G., Quach C. (2022). Hand hygiene compliance in the prevention of hospital-acquired infections: A systematic review. J. Hosp. Infect..

[31] World Health Organization (2023). Global Strategy on Infection Prevention and Control. https://cdn.who.int/media/docs/default-source/gsipc/who_ipc_global-strategy-for-ipc.pdf?sfvrsn=ebdd8376_4.

[32] Pietrzak J.R.T., Geldenhuys D.B., Sekeitto A.R., Sikhauli N., Mokete L. (2026). Clean Air, Safe Joints: Optimizing Operating Theatre Air Quality to Reduce Surgical Site and Periprosthetic Joint Infections. Orthop. Rev..

[33] Koota E., Kaartinen J., Melender H.L. (2024). Impact of educational interventions for professionals on infection control practices to reduce healthcare-associated infections and prevent infectious diseases: A systematic review. Collegian.

[34] Sartelli M., Marini C.P., McNelis J., Coccolini F., Rizzo C., Labricciosa F.M., Petrone P. (2024). Preventing and Controlling Healthcare-Associated Infections: The First Principle of Every Antimicrobial Stewardship Program in Hospital Settings. Antibiotics.

[35] Tanner W.D., Leecaster M.K., Zhang Y., Stratford K.M., Mayer J., Visnovsky L.D., Alhmidi H., Cadnum J.L., Jencson A.L., Koganti S. (2021). Environmental Contamination of Contact Precaution and Non-Contact Precaution Patient Rooms in Six Acute Care Facilities. Clin. Infect. Dis..

[36] Center for Disease Control and Prevention (2023). Summary of Recommendations. Guideline for Isolation Precautions: Preventing Transmission of Infectious Agents in Healthcare Settings. https://www.cdc.gov/infection-control/hcp/isolation-precautions/summary-recommendations.html.

[37] Fischer D., Schlößer R.L., Kempf V.A.J., Wichelhaus T.A., Klingebiel T., Philippi S., Falgenhauer L., Imirzalioglu C., Dahl U., Brandt C. (2019). Overcrowding in a neonatal intermediate care unit: Impact on the incidence of multidrug-resistant gram-negative organisms. BMC Infect. Dis..

[38] Ogunsola F.T., Mehtar S. (2020). Challenges regarding the control of environmental sources of contamination in healthcare settings in low-and middle-income countries—A narrative review. Antimicrob. Resist. Infect. Control.

[39] Mitchell B.G., Gardner A., Stone P.W., Hall L., Pogorzelska-Maziarz M. (2018). Hospital Staffing and Health Care-Associated Infections: A Systematic Review of the Literature. Jt. Comm. J. Qual. Patient Saf..

[40] Bartles R., Reese S., Gumbar A. (2024). Closing the gap on infection prevention staffing recommendations: Results from the beta version of the APIC staffing calculator. Am. J. Infect. Control.

[41] Nadi Z.B., Raisali F., Jafari N., Bayramzadeh S. (2024). The influence of physical environment on health care-associated infections: A literature review. Am. J. Infect. Control.

[42] Prasek K., Kiersnowska I., Wójkowska-Mach J., Różańska A., Romaniszyn D., Foryciarz E., Kwiećkowska L.B., Krzych-Fałta E. (2025). Microbial Contamination on High-Touch Surfaces in Outpatient Clinics: Identification of Bacterial Strains from Areas of Patient and Medical Staff Occupancy. Microorganisms.

[43] Shams A.M., Rose L.J., Edwards J.R., Cali S., Harris A.D., Jacob J.T., LaFae A., Pineles L.L., Thom K.A., McDonald L.C. (2016). Assessment of the Overall and Multidrug-Resistant Organism Bioburden on Environmental Surfaces in Healthcare Facilities. Infect. Control Hosp. Epidemiol..

[44] Cobrado L., Silva-Dias A., Azevedo M.M., Rodrigues A.G. (2017). High-touch surfaces: Microbial neighbours at hand. Eur. J. Clin. Microbiol. Infect. Dis..

[45] Nas M.Y., Ibiebele J., Dolgin G., Malczynski M., Qi C., Bolon M., Zembower T. (2020). The intersection of hand hygiene, infusion pump contamination, and high alarm volume in the health care environment. Am. J. Infect. Control..

[46] Pinheiro-Hubinger L., Moraes Riboli D.F., Abraão L.M., Pereira Franchi E.P.L., Ribeiro de Souza da Cunha M.d.L. (2021). Coagulase-Negative Staphylococci Clones Are Widely Distributed in the Hospital and Community. Pathogens.

[47] Bhandari R., Amatya N.M., Bogati P., Sulu P., Baniya P. (2025). Unveiling Hidden Threats: Bacterial Contamination of Frequently Touched Objects and the Biofilm Property of *Staphylococcus aureus* as a Threat to Antibiotic Success. Can. J. Infect. Dis. Med. Microbiol..

[48] Harsent R., Cattoir V., Pascoe M., Pertusati F., Westwell A.C., Maillard J.Y. (2026). *Enterococcus* spp. ability to form a dry surface biofilm: A route to persistence on environmental surfaces. J. Hosp. Infect..

[49] Fahy S., O’Connor J.A., Lucey B., Sleator R.D. (2023). Hospital Reservoirs of Multidrug Resistant *Acinetobacter* Species-The Elephant in the Room!. Br. J. Biomed. Sci..

[50] Ristori M.V., Scarpa F., Sanna D., Casu M., Petrosillo N., Longo U.G., Lucia F., Spoto S., Chiantia R.M., Caserta A. (2024). Multidrug-Resistant *Klebsiella pneumoniae* Strains in a Hospital: Phylogenetic Analysis to Investigate Local Epidemiology. Microorganisms.

[51] Virieux-Petit M., Ferreira J., Masnou A., Bormes C., Paquis M.P., Toubiana M., Bonzon L., Godreuil S., Romano-Bertrand S. (2025). Assessing the role of environment in *Pseudomonas aeruginosa* healthcare-associated bloodstream infections: A one-year prospective survey. J. Hosp. Infect..

[52] Prigitano A., Perrone P.M., Esposto M.C., Carnevali D., De Nard F., Grimoldi L., Principi N., Cogliati M., Castaldi S., Romanò L. (2022). ICU environmental surfaces are a reservoir of fungi: Species distribution in northern Italy. J. Hosp. Infect..

[53] Ghodsi S., Nikaeen M., Aboutalebian S., Mohammadi R., Mirhendi H. (2025). Prevalence of fungi and their antifungal and disinfectant resistance in hospital environments: Insights into combating nosocomial mycoses. Antimicrob. Resist. Infect. Control.

[54] Breijyeh Z., Jubeh B., Karaman R. (2020). Resistance of Gram-Negative Bacteria to Current Antibacterial Agents and Approaches to Resolve It. Molecules.

[55] Maher C., Hassan K.A. (2023). The Gram-negative permeability barrier: Tipping the balance of the in and the out. mBio.

[56] Boretti A., Banik B. (2025). Antibiotic Resistance: Revisiting Older Antibiotics for Modern Bacterial Challenges. Chem. Biodivers..

[57] Noshak M.A., Rezaee M.A., Hasani A., Mirzaii M. (2020). The Role of the Coagulase-negative Staphylococci (CoNS) in Infective Endocarditis; A Narrative Review from 2000 to 2020. Curr. Pharm. Biotechnol..

[58] Facciolà A., Laganà A., Gioffrè M.E., Morabito A., Chiera D., Ferlazzo M., Laganà P. (2025). Staphylococci: What Has Changed in the Antibiotic Resistance Profile in the Last Decade-Analysis of Strains Isolated from Hospitalised Patients. Pathogens.

[59] González-Alonso M., Lajara-Heredia A., Guerra-González A., Villar-Suárez V., Sánchez-Lázaro J.A. (2026). Microbiological Patterns in Periprosthetic Knee Infections over a Decade: Analysis of Resistance Patterns, Temporal Trends, and Patient Residence. Antibiotics.

[60] França A. (2023). The Role of Coagulase-Negative Staphylococci Biofilms on Late-Onset Sepsis: Current Challenges and Emerging Diagnostics and Therapies. Antibiotics.

[61] Crepin D.M., Chavignon M., Verhoeven P.O., Laurent F., Josse J., Butin M. (2024). *Staphylococcus capitis*: Insights into epidemiology, virulence, and antimicrobial resistance of a clinically relevant bacterial species. Clin. Microbiol. Rev..

[62] Liu C., Yu J., Chen C., Li X., Ye Y., Dong Y., Ying X., Li H., Wang W. (2024). Characterization of linezolid- and methicillin-resistant coagulase-negative staphylococci in a tertiary hospital in China. BMC Infect. Dis..

[63] Tao S., Chen H., Li N., Wang T., Liang W. (2022). The Spread of Antibiotic Resistance Genes In Vivo Model. Can. J. Infect. Dis. Med. Microbiol..

[64] Michaelis C., Grohmann E. (2023). Horizontal Gene Transfer of Antibiotic Resistance Genes in Biofilms. Antibiotics.

[65] Almeida-Santos A.C., Novais C., Peixe L., Freitas A.R. (2025). Vancomycin-resistant *Enterococcus faecium*: A current perspective on resilience, adaptation, and the urgent need for novel strategies. J. Glob. Antimicrob. Resist..

[66] Radford-Smith D.E., Anthony D.C. (2025). Vancomycin-Resistant *E. faecium*: Addressing Global and Clinical Challenges. Antibiotics.

[67] Neely A.N., Maley M.P. (2000). Survival of enterococci and staphylococci on hospital fabrics and plastic. J. Clin. Microbiol..

[68] Kitagawa H., Tadera K., Mori M., Kashiyama S., Nomura T., Omori K., Shigemoto N., Ohge H. (2021). The effect of pulsed-xenon ultraviolet disinfection on surfaces contaminated with vancomycin-resistant Enterococci in a Japanese hospital. J. Infect. Chemother..

[69] Shamsizadeh Z., Nikaeen M., Nasr Esfahani B., Mirhoseini S.H., Hatamzadeh M., Hassanzadeh A. (2017). Detection of antibiotic resistant *Acinetobacter baumannii* in various hospital environments: Potential sources for transmission of *Acinetobacter* infections. Environ. Health Prev. Med..

[70] Borges Duarte D.F., Gonçalves Rodrigues A. (2022). *Acinetobacter baumannii*: Insights towards a comprehensive approach for the prevention of outbreaks in health-care facilities. APMIS.

[71] Karampatakis T., Tsergouli K., Behzadi P. (2023). Carbapenem-Resistant *Klebsiella pneumoniae*: Virulence Factors, Molecular Epidemiology and Latest Updates in Treatment Options. Antibiotics.

[72] Leistner R., Kohlmorgen B., Brodzinski A., Schwab F., Lemke E., Zakonsky G., Gastmeier P. (2023). Environmental cleaning to prevent hospital-acquired infections on non-intensive care units: A pragmatic, single-centre, cluster randomized controlled, crossover trial comparing soap-based, disinfection and probiotic cleaning. EClinicalMedicine.

[73] Lopez-Alcalde J., Mateos-Mazón M., Oliveira Conterno L., Dancer S.J., Stallings E.C., Villanueva G., Gonzalez-Velez A.E., Cabir Nunes S., Jiménez-Tejero E., Solà I. (2026). Decontamination of environmental surfaces in hospitals to reduce hospital-acquired infections: A systematic review. Infect. Prev. Pract..

[74] Tong C., Hu H., Chen G., Li Z., Li A., Zhang J. (2021). Disinfectant resistance in bacteria: Mechanisms, spread, and resolution strategies. Environ. Res..

[75] Rozman U., Pušnik M., Kmetec S., Duh D., Šostar Turk S. (2021). Reduced Susceptibility and Increased Resistance of Bacteria against Disinfectants: A Systematic Review. Microorganisms.

[76] Denkel L.A., Voss A., Caselli E., Dancer S.J., Leistner R., Gastmeier P., Widmer A.F. (2024). Can probiotics trigger a paradigm shift for cleaning healthcare environments? A narrative review. Antimicrob. Resist. Infect. Control.

[77] Falagas M.E., Kontogiannis D.S., Sargianou M., Falaga E.M., Chatzimichali M., Michaeloudes C. (2025). Probiotic-Based Cleaning Solutions: From Research Hypothesis to Infection Control Applications. Biology.

[78] Sahoo J., Sarkhel S., Mukherjee N., Jaiswal A. (2022). Nanomaterial-Based Antimicrobial Coating for Biomedical Implants: New Age Solution for Biofilm-Associated Infections. ACS Omega.

[79] Laganà A., Visalli G., Corpina F., Ferlazzo M., Di Pietro A., Facciolà A. (2023). Antibacterial activity of nanoparticles and nanomaterials: A possible weapon in the fight against healthcare-associated infections. Eur. Rev. Med. Pharmacol. Sci..

[80] Laganà A., Facciolà A., Iannazzo D., Celesti C., Polimeni E., Biondo C., Di Pietro A., Visalli G. (2023). Promising Materials in the Fight against Healthcare-Associated Infections: Antibacterial Properties of Chitosan-Polyhedral Oligomeric Silsesquioxanes Hybrid Hydrogels. J. Funct. Biomater..

[81] Puertas-Segura A., Laganà A., Rathee G., Savage P., Todorova K., Dimitrov P., Pashkuleva I., Reis R.L., Ciardelli G., Tzanov T. (2025). Ceragenin Nanogel Coating Prevents Biofilms Formation on Urinary Catheters. ACS Appl. Mater. Interfaces.

